# Predicting active school travel: The role of planned behavior and habit strength

**DOI:** 10.1186/1479-5868-9-65

**Published:** 2012-05-30

**Authors:** Shemane Murtagh, David A Rowe, Mark A Elliott, David McMinn, Norah M Nelson

**Affiliations:** 1 Physical Activity for Health Research Group, School of Psychological Sciences and Health, University of Strathclyde, Glasgow, UK; 2 Traffic and Transport Psychology Group, School of Psychological Sciences and Health, University of Strathclyde, Glasgow, UK; 3 Rowett Institute of Nutrition and Health, School of Medicine and Dentistry, University of Aberdeen, Aberdeen, UK; 4 School of Culture and Lifestyle, University of Derby, Buxton, UK

**Keywords:** Theory of planned behavior, Habit, Active school travel, Walking, Children

## Abstract

**Background:**

Despite strong support for predictive validity of the theory of planned behavior (TPB) substantial variance in both intention and behavior is unaccounted for by the model’s predictors. The present study tested the extent to which habit strength augments the predictive validity of the TPB in relation to a currently under-researched behavior that has important health implications, namely children’s active school travel.

**Method:**

Participants (N = 126 children aged 8–9 years; 59 % males) were sampled from five elementary schools in the west of Scotland and completed questionnaire measures of all TPB constructs in relation to walking to school and both walking and car/bus use habit. Over the subsequent week, commuting steps on school journeys were measured objectively using an accelerometer. Hierarchical multiple regressions were used to test the predictive utility of the TPB and habit strength in relation to both intention and subsequent behavior.

**Results:**

The TPB accounted for 41 % and 10 % of the variance in intention and objectively measured behavior, respectively. Together, walking habit and car/bus habit significantly increased the proportion of explained variance in both intention and behavior by 6 %. Perceived behavioral control and both walking and car/bus habit independently predicted intention. Intention and car/bus habit independently predicted behavior.

**Conclusions:**

The TPB significantly predicts children’s active school travel. However, habit strength augments the predictive validity of the model. The results indicate that school travel is controlled by both intentional and habitual processes. In practice, interventions could usefully decrease the habitual use of motorized transport for travel to school and increase children’s intention to walk (via increases in perceived behavioral control and walking habit, and decreases in car/bus habit). Further research is needed to identify effective strategies for changing these antecedents of children’s active school travel.

## Background

Physical activity in childhood is associated with a range of health benefits including a reduced risk of cardiovascular disease [1] and obesity [2], and improved mental wellbeing [3]. However, in Scotland, 19 % of girls and 11 % of boys meet the current recommended minimum target of at least one hour of physical activity per day [4]. The transition from childhood into early adolescence is a key developmental period during which physical activity notably decreases [5] and the promotion of active travel (e.g., walking) has been identified as a means for helping children to maintain physical activity and establish lifelong health habits [6]. While interventions to promote active travel have been implemented over the last decade, they have had only small or non-significant effects on behavior [7]. Two possible explanations are that interventions have been developed without a theoretical basis [7] and active travel is strongly governed by habits, which are notoriously difficult to change [8]. Research that identifies theoretically derived predictors of children’s active travel and takes into account the effects of habituation is therefore required. The present study addresses these issues by providing the first test of the theory of planned behavior (TPB; [9]) and habit strength in the context of children’s active school travel.

### The Theory of Planned Behavior

The TPB is a model of rational decision-making which proposes that behavior is determined by a number of potentially changeable cognitions. It is therefore a suitable model for helping researchers to identify targets for health interventions [10]. The model proposes that intention is the proximal determinant of behavior. Intentions are indications of how much a person wants to perform a behavior and how hard they are willing to try in order to perform it [9]. Intentions are, in turn, determined by three constructs; attitude, subjective norm and perceived behavioral control. Attitude is an overall positive or negative evaluation about performing the behavior and comprises both an affective (enjoyable vs. unenjoyable) and an instrumental (harmful vs. beneficial) component [11]. Subjective norm also comprises two related components: descriptive norm refers to an individual’s perception about how often important social referents (e.g., friends) will perform the behavior; injunctive norm refers to how much an individual thinks that important social referents would want him or her to perform the behavior [12]. Finally, perceived behavioral control reflects a person’s perceived ability to perform a behavior. In addition to being an independent predictor of intention it is held to predict behavior directly, along with intention, so long as it reflects the actual control an individual has over their behavior [9,13]. Perceived behavioral control therefore helps predict behaviors that are not under complete volitional control [9]. This makes the TPB a potentially suitable model for understanding children’s active school travel behavior, since this behavior is not only likely to be influenced by motivation (e.g., attitudes and normative pressure to walk to school) but also external constraints (e.g., parental control over mode of school travel [14]).

Although the TPB has not been applied previously to children’s active travel, it has been found to predict related behaviors such as travel mode choices in adults [15] and non-travel related physical activity in both adults and children [16]. In line with meta-analytic reviews of studies on general social behavior [17,18] this research shows that the TPB accounts for large proportions of variance (R^2^ > .25; see Cohen [19]) in both intention and behavior. However, despite the support for the TPB, the evidence base is largely characterized by cross-sectional designs and the use of subjective (self-reported) behavior measures, both of which represent methodological limitations. Cross-sectional designs have been commonly used in studies of general physical activity [20] and travel choices [15], although it is acknowledged that a small number of studies have provided prospective tests of the TPB in these domains (e.g., [21]). More generally cross-sectional designs are employed in over half of all published TPB studies [22]. They are problematic because the contemporaneous measurement of TPB constructs and behavior means that behavior measures are indications of past behavior, and predicting past behavior from TPB constructs violates the causal ordering proposed by the model (e.g., intention → behavior) and creates a confound when researchers examine the effects of habit on behavior. With respect to subjective behavior measures, a recent meta-analysis by McEachan et al. [18] found that, of 237 tests of the TPB, behavior was assessed using self-report methods (rather than objective observations) in 86% of cases. Self-reported behavior measures are vulnerable to cognitive [23], affective [24] and self-presentational [25] biases, which can lead to inaccuracies in behavior data (i.e., under- or over-reporting). In support of this contention, research examining both self-reported and objectively measured walking behavior (e.g., assessed through pedometers) has found no association between the two [26] and Adamo et al. [27] reported that children substantially over-estimate their physical activity levels (by 114% for boys and 584% for girls, on average). In the context of active travel therefore self-reported behavior measures may lack validity. Also, the TPB has been shown to account for more variance in subjectively measured behavior than objectively measured behavior, which could be due, in part, to common method variance between TPB and subjective behavior measures [17,28]. For these reasons we used a prospective measure of observed (i.e., objective) behavior in this first application of the TPB to children’s active school travel and consistent with previous research on children’s general physical activity [29,30], we used self-report questionnaires to measure TPB constructs. Therefore, in line with the TPB we hypothesized that attitude, subjective norm, and perceived behavioral control would together account for a significant proportion of the variance in children’s intentions to walk to school and that intention and perceived behavioral control would together account for a significant proportion of the variance in a prospective measure of objective behavior.

### Habit

We also tested the extent to which habit augments the predictive validity of the TPB. Habits are learned patterns of behavior that are initiated automatically in response to situational cues and they are formed when behavior is performed frequently in stable contexts [31]. Children’s school travel behavior is therefore likely to be under habitual control, at least to some extent, because journeys to school are characterized by both repetition (i.e., they are typically made each day of the school week) and situational stability (i.e., they take place at approximately the same time of day, have the same start and end points and typically constitute the same route). However, while several studies have shown that habit accounts for additional variance in both intention and behavior, over and above the variance that is accounted for by the TPB [32,33], none of these studies has been concerned with children’s active school travel, and studies on related behaviors (e.g., general physical activity or travel choices) have tended to use self-reported behavior measures or cross sectional designs [34,35]. Given also that reliable and valid measures of habit can be obtained from children in the present target age group using self-completion questionnaires [36-38], we tested the extent to which school travel habits increase the proportion of explained variance in both intention and active school travel behavior.

Finally, we also tested the extent to which habit moderates the intention-behavior relationship. No previous study has tested this moderation effect in the context of children’s active travel and research in other domains has provided mixed evidence. Several studies have shown that the effects of intention and habit on behavior are independent [35,39]. Other studies have demonstrated a moderation effect but, as highlighted by Ajzen [40], some have shown that the effect of intention on behavior decreases with habit and others have shown that the effect of intention increases with habit. The former moderation effect is consistent with the traditional behaviorist view that rational decision-making (e.g., intention) does not influence behavior when under the influence of habit because behavioral control is turned over to situational cues (i.e., behavior is, in effect, carried out without conscious thought). The latter moderation effect is in keeping with a cognitive-motivational account of habit, which views habits as goal (i.e., intention) serving and thus places greater emphasis on decision-making processes in the execution of behavior [41]. Identifying whether habit moderates the intention-behavior relationship, and the direction of any moderating effect is therefore of theoretical importance (i.e., for understanding the interplay between intentional and habitual control of behavior). It also has important practical value because it sheds light on the conditions under which health interventions, designed to change intentions (e.g., to actively travel to school), might have the scope to promote behavior change.

### Study aims and hypotheses

In the present investigation the aim was to investigate the predictive utility of the TPB and the role of habit in relation to children’s active school travel. In line with the above background, we hypothesized the following. First, the TPB would account for a significant proportion of the variance in children’s intentions to walk to school and their objectively measured active school travel behavior. Second, habit would account for additional variance in both intention and behavior. Finally, given the mixed evidence for the moderating effect of habit on the intention-behavior relationship we specified no related hypothesis for the third research question.

## Method

### Participants

Participants were 126 children sampled from five elementary schools in Glasgow (a large city in the west of Scotland, UK). All participants were aged between 8 and 9 years old (M = 8.66, SD = 0.49) and 59 % of the sample was male.

### Design and procedure

This study formed part of the project: Strathclyde Evaluation of Children’s Active Travel (SE-CAT). This larger project was designed to evaluate the effectiveness of a school-based intervention aimed at encouraging children to walk and cycle to school. This article reports an analysis of the pre-intervention data, which were not contaminated by any potential intervention effects and therefore allowed us to test our stated hypotheses. Ethical approval for the study was granted by the University of Strathclyde Research Ethics Committee.

The data reported in this article were collected during the fall school term (Sept-Nov 2009).

Following permissions granted by Local Education Authorities, five elementary schools agreed to take part in the research. These schools provided a broad coverage of socio-economic status, with three of the schools being located in the highest ranked quintile of deprived areas in Glasgow and two of the schools being located in the lowest ranked quintile of deprived areas. In each school, study information sheets and consent forms were distributed to 232 children and their parents. Signed parent and child consent forms were obtained for 167 participants (a 72 % consent rate). To ensure that walking was a viable mode of school travel, children living more than three miles away from their school (n = 41) were not included in the present analyses.

At each school, data collection involved issuing self-completion questionnaires to children at the beginning (Monday) of the school week. Each child completed one questionnaire as part of a 1-hour class. The questionnaires measured all constructs from the TPB, operationalized with respect to ‘walking to school every day’, and habit strength, operationalized with respect to both walking to school (walking habit) and traveling to school via car or bus (car/bus habit)^1^. The questionnaires were administered by teams of 4–5 trained research assistants who provided the children with help understanding the questions, if needed.

After completing the questionnaires all participants were fitted with an accelerometer (Actigraph GT1M, Pensacola, FL) for the remainder of the school week. The accelerometers were attached to the participants’ right hip with an elastic belt. Children were instructed to wear the belts from first thing in the morning, after waking, until last thing at night, before going to bed. They were also told that the belts should only be taken off when bathing, showering or swimming. The accelerometer data allowed subsequent active travel behavior (step count) to be measured objectively on four morning commutes to school (from 5.30 am until school arrival time on Tuesday to Friday) and four afternoon commutes home (from 3.00 pm until arrival at home on Monday to Thursday). Accelerometers have been extensively used to measure physical activity, and have been validated for use in children against indirect calorimetry in both laboratory [42] and free-living [43] conditions. Although reactivity is always a concern with any measurement tool, studies examining pedometer reactivity in children have concluded that no reactivity exists [26,44].

### Measures

#### Theory of Planned Behavior constructs

Standard measures of all TPB constructs were used following guidelines provided by Fishbein and Ajzen [45]. However, the wording of all questionnaire items and the response options was amended following previous research on children in the present age range [39,40]. Participants responded to all items using 4-point scales, scored 1 (disagree in a big way), 2 (disagree), 3 (agree) or 4 (agree in a big way).

Intention was measured using two items: ‘I plan to walk to school every day’ and ‘I intend to walk to school every day’. The mean of participants’ scores on these two items served as the overall measure of intention for use in the subsequent data analysis (α = .84). Attitude was measured with four items. Consistent with the distinction in the literature [11] two items tapped into the affective component of this construct (‘Walking to school every day would be fun’ and ‘Walking to school every day would be enjoyable’) and two items tapped into the instrumental component (‘Walking to school every day would be good for me’ and ‘Walking to school every day would be important for me’). The mean of the four items served as the overall measure of attitude for use in the subsequent analyses (α = .74). Similarly, the mean of six items served as the measure of subjective norm (α = .71). Three of these items measured the injunctive component of subjective norm and three items measured the descriptive component [11,12]. The three injunctive items were: ‘My family wants me to walk to school every day’; ‘My friends want me to walk to school every day’; and ‘My teachers want me to walk to school every day’. The three descriptive items were: ‘My family will walk to school or work every day’; ‘My friends will walk to school every day’; and ‘My teachers will walk to school every day’. Finally, perceived behavioral control was measured with three items: ‘I could walk to school every day if I wanted to’; ‘I have the time to walk to school every day if I wanted to’; and ‘I live in a place which allows me to walk to school every day if I wanted to’. The mean of these three items was calculated for each participant and served as the final measure of perceived behavioral control (α = .75).

#### Habit

The Self-Report Habit Index (SRHI; [46] was used to measure both walking and car/bus habit. The SRHI comprises 12 items which together tap into three key features of habit; that is, frequency of past behavior (e.g., behavior X is something ‘I do frequently’), behavioral automaticity (e.g., behavior X is something ‘I do automatically’) and identity expression (e.g., behavior X is something ‘that is typically me’). It therefore provides a conceptually stronger measure of habit than is provided by measures of past behavior, which are used in many studies to measure habit [31]. Additionally, the reliability and validity of the SRHI as a measure of habit has been previously established in research on both adults [46] and children [36-38]. In the present study, participants completed the 12 SRHI items with respect to both “Walking to school” and “Traveling by car or bus to school”. Responses were recorded on 5-point Likert scales scored from 1 (totally disagree) to 5 (totally agree). The mean of the 12 walking habit items (α = .94) and the mean of the 12 car/bus habit items (α = .97) served as the final measures of walking and car/bus habit, respectively.

#### Subsequent behavior

Accelerometer data collected over the week following questionnaire administration were used to derive the objective measure of subsequent behavior (active school travel). For each participant, the mean number of steps across the eight commutes to and from school was calculated (α = .87). This provided a measure of the average number of steps per school commute, for each participant.

#### Data analysis

The data were analysed in SPSS (version 18; IBM Corp., Chicago, IL) using techniques that are commonly employed in TPB research, namely correlation and multiple regression.

## Results

### Descriptive statistics

Descriptive statistics for all TPB constructs, walking and car/bus habit, and subsequent behavior are provided in Table 1. Participants, on average, had positive attitudes and intentions towards walking to school, perceived social pressure (subjective norm) to walk to school and perceived that they had control over their performance of this behavior (i.e., the sample means for these constructs were above the scale mid-point, 2.5). Additionally, participants, on average, reported moderate levels of walking habit (i.e., the sample mean was slightly above the scale mid-point, 3) and low levels of car/bus habit. The average number of steps per school commute (behavior) was 2,262.

**Table 1:** Means, standard deviations and zero order correlations for each study variable (n= 126)

**Variables**	**M**	**SD**	**1**	**2**	**3**	**4**	**5**	**6**	**7**
1. Commuting steps	2262.05	1006.37	-	.31**	.19*	.15	.15	.15	-.36**
2. Intention ^a^	3.12	0.77		-	.64**	.35**	.40**	.38**	-.45**
3. Perceived Behavioral Control ^a^	3.25	0.67			-	.52**	.48**	.26**	-.44**
4. Attitude ^a^	3.42	0.49				-	.37**	.22*	-.18*
5. Subjective Norm ^a^	2.69	0.55					-	.36**	-.22*
6. Walking Habit ^b^	3.53	1.20						-	-.33**
7. Car/bus Habit ^b^	2.35	1.34							-

Note. ^a^Range of possible scores = 1-4, with higher scores indicating more positive intentions to walk to school, greater perceptions of control, more positive attitudes and greater perceived normative pressure (subjective norm). ^b^Range of possible scores = 1 - 5, with higher scores indicating higher levels of walking and car/bus habit.* p < .05.** p < .01.

### Zero order correlations

In line with the TPB, the zero order correlations in Table 1 show that both intention and perceived behavioral control were positively correlated with behavior and attitude, subjective norm and perceived behavioral control were each positively correlated with intention. Also in line with the predictions, walking habit was positively correlated with intention (but not behavior) and car/bus habit was negatively correlated with both intention and behavior. The directions of these correlations show that intention to walk to school increased with attitude, subjective norm, perceived behavioral control and walking habit, and decreased with car/bus habit. Behavior increased with intention and perceived behavioral control, and decreased with car/bus habit but was uncorrelated with walking habit.

### Prediction of intentions

A hierarchical multiple linear regression was used to test the predictive validity of the TPB and habit in relation to intention (see Table 2). At step 1, the independent variables were attitude, subjective norm and perceived behavioral control. Walking and car/bus habit were entered as additional independent variables at step 2. At step 1, the three TPB constructs together accounted for 41% of the variance in intention (R^2^ = .41, F_(3, 122)_ = 28.77, p < .01). Perceived behavioral control was an independent predictor at step 1 (β = .58, p < .01), but attitude (β = .00, p > .05), and subjective norm (β = .12, p > .05) were not. At step 2, the addition of walking and car/bus habit accounted for a 6% increase in explained variance in intention (R^2change^ = .06, F_change (2, 120)_ = 6.66, p < .01). Walking habit (β = .18, p < .05) and car/bus habit (β = −.16, p < .05) were both independent predictors of intention at step 2 and perceived behavioral control remained an independent predictor (β = .49, p < .01).

**Table 2:** Hierarchical multiple regression predicting intention to walk to school from TPB constructs (step 1), and walking and car/bus use habit (step 2)

**Step**	**Variables Entered**	**R^2^**	**R^2change^**	**F change**	**β_Step1_**	**β_Step2_**
1.	Attitude	.41	.41	28.77**	.00	.00
	Subjective Norm				.12	.06
	Perceived Behavioral Control				.58**	.49**
2.	Walking Habit	.47	.06	6.66**		.18*
	Car/Bus Habit					-.16*

* p < .05. ** p < .01.

### Prediction of behavior

Another hierarchical multiple linear regression was used to test the predictive validity of the TPB and habit in relation to objectively measured subsequent behavior. Intention and perceived behavioral control (i.e., the direct predictors of behavior according to the theory) were entered at step 1 of the analysis and walking and car/bus habit were both entered at step 2. As can be seen in Table 3, intention and perceived behavioral control together accounted for 10% of the variance in behavior (R^2^ = .10, F_(2,123)_ = 6.44, p < .01). Intention was the sole independent predictor (β = .32, p < .01). When added to the analysis at step 2, the habit measures increased the explained variance in behavior by 6% (R^2change^ = .06, F_change (2, 121)_ = 4.61, p < .01). Car/bus habit independently predicted behavior (β = −.29, p < .01) at step 2 but walking habit did not (β = −.02, p > .05). Intention remained a significant predictor of behavior at step 2 (β = .24, p < .05). Also reported in Table 3 are the effects of two-way interactions between intention and walking habit and intention and car/bus habit. Following standard procedures [43], these interactions were included at step 3 of the regression analysis to test the moderating role of habit on the intention-behavior relationship. Before the two-way interactions were calculated, intention and both walking and car/bus habit were mean centered to reduce the possible effects of multicollinearity [44]. As can be seen in Table 3, the interaction terms did not account for any additional variance in behavior, over and above the variance accounted for by the TPB and habit measures (R^2change^ = .00, F_change (2, 119)_ = .16, p > .05). Neither interaction independently predicted behavior.

**Table 3:** Hierarchical multiple regression predicting active school travel behavior (commuting steps) from TPB constructs (step 1), walking and car/bus use habit (step 2), and intention X habit interactions (step 3)

**Step**	**Variables Entered**	**R^2^**	**R^2change^**	**F change**	**β_Step1_**	**β_Step2_**	**β_Step3_**
1.	Intention	.10	.10	6.44**	.32**	.24*	.24*
	Perceived Behavioral Control				-.01	-.09	-.10
2.	Walking Habit	.16	.06	4.61**		-.02	-.01
	Car/Bus Habit	.47	.06	6.66**		-.29**	-.29**
3.	Intention X Walking Habit	.16	.00	0.16			.05
	Intention X Car/Bus Habit						.01

* p < .05. ** p < .01.

## Discussion

This study represents the first test of the TPB and the effects of habit in relation to children’s active school travel. It therefore offers an important contribution to knowledge, providing new insights into the psychological antecedents of this behavior - antecedents which are likely to constitute useful targets for theory-based health interventions (e.g., educational programs that aim to promote walking to school). The following subsections address the support provided for the TPB, the effects of habit on children’s active school travel and the implications of the findings for developing effective health interventions.

### Support for the Theory of Planned Behavior

Overall, the results support the first hypothesis because they demonstrate the predictive validity of the TPB in the context of children’s active school travel. First, the model accounted for 41% of the variance in children’s intentions to walk to school. This is regarded as a large-sized effect in the social sciences [19] and compares well with previous findings from TPB studies on other behaviors, which also show that the model accounts for large proportions of variance in intentions [15-18]. Second, the model accounted for 10% of the variance in active school travel behavior. Although research on the TPB generally demonstrates somewhat larger effects on behavior [15-18], the present finding is still regarded as a moderate-sized effect in the social sciences [19] and is encouraging given that we provided a rigorous test of the TPB, using an objective measure of subsequent behavior.With respect to the independent effects of the TPB constructs, perceived behavioral control was an independent predictor of children’s intentions to actively travel to school. Intention, in turn, was an independent predictor of subsequent behavior. These effects also remained statistically significant after controlling for the effects of habit. On the other hand, neither attitude nor subjective norm independently predicted intention, and perceived behavioral control did not predict behavior directly. While these relationships are posited by the TPB, it is expected that the relative importance of the model’s constructs will vary across different behaviors, contexts and populations, and not all components will be needed to predict intentions and behavior in all cases [9]. Indeed, there is variation in the independent effects of TPB constructs across different studies [47]. Thus, the present null results do not necessarily refute the TPB as a model of behavior. Instead, the present findings imply that, of the cognitive variables proposed by the model, all that is needed to predict intention to walk to school (in children aged 8–9 years old) is perceived behavioral control and all that is needed to predict behavior is intention. A potential explanation for the lack of prediction from attitude and subjective norm is that, for children in the present age range, school travel is not under complete volitional control, with many parents not permitting their children to walk to school (e.g., due to perceptions that it is unsafe to do so; see Granville et al [48]). In contrast, perceived control takes into account such constraints upon behavior and is therefore equipped to predict non-volitional behaviors [9]. That said, the finding that intention predicted behavior but perceived control did not suggests that for active school travel, perceived control serves to influence motivation but does not influence behavior. A possible explanation is that for the focal behavior in this study, parental control over decision-making may be more critical, and the measure of perceived control used in this study does not explicitly address parental influences on children’s actual control over the decision to commute actively. Further research is therefore needed to examine the accuracy of children’s perceptions of control over behavior in the context of active school travel, and the role of parental decision-making control.

### The role of habit

In addition to demonstrating the importance of reasoned-decision making (i.e., TPB constructs), the present findings demonstrate the importance of habit in the prediction of children’s active school travel behavior. In line with the second hypothesis, walking and car/bus habit together increased the prediction of both intention and behavior, over and above the TPB. These findings are consistent with previous studies that also support the role of habituation in the execution of behavior, including studies of travel mode choices [15] and non-travel related physical activity [16,49]. Importantly however, the present findings represent an important contribution to the literature because the effects of habit were assessed prospectively, using an objective behavior measure that is not susceptible to self-reporting biases.

It is also worth noting that both walking and car/bus habit were found to independently predict children’s intentions to actively travel to school. The implications are that intentions to walk to school are, in part, automatically formed (i.e., on the basis of habits) and being in the habit of walking to school and (not) being in the habit of traveling to school by car or bus serve to motivate behavior separately. On the other hand, only car/bus habit was found to predict behavior directly, showing that this habit also has a more proximal effect on behavior, which is independent of the effect of intention. That is not to say that habitual and rational decision-making are in competition. In fact, the present findings show that neither measure of habit moderates the intention-behavior relationship. Thus, the findings do not support the behaviorist view that habit diminishes the effects of rational decision-making on behavior. At the same time, the lack of a moderation effect provides little support for the social cognitive view that habits are goal-serving [41]. In the present context, the findings suggest that intentional and habitual processes have complementary but essentially independent effects on active school travel behavior. This supports previous studies in other domains which have also failed to find significant interactions between intention and habit [35,39].

### Practical implications

From a practical point of view the present findings suggest that interventions could usefully increase children’s perception of control over their ability to walk to school, promote walking habits and reduce car/bus habits (i.e., these variables were significant predictors of intentions to walk to school and intentions, in turn, predicted behavior). In particular, interventions that successfully reduce the habit of traveling to school by motorized transport are likely to be particularly effective given that car/bus habit not only predicted intention but also behavior directly.

Effective ways to increase perceived behavioral control are well established [50] and include the promotion of personal mastery experiences (e.g., successful performance of a behavior following guidance, sub-tasking or visualization), vicarious experiences (observing and then modeling the required behavior), verbal persuasion (e.g., immediate positive feedback following successful behavioral performance), and emotional arousal (e.g., stressing the benefits of performing a behavior and the risks of not doing so). In school settings such techniques have been found to increase children’s academic performance [51,52]. It is possible therefore that these techniques will also be effective for promoting other behaviors in children. Future research is needed to test the effectiveness of these techniques for promoting active school travel.

Techniques to change habits have received less research attention. However, according to the habit discontinuity hypothesis [53], the dependence of habits on environmental cues can provide an opportunity to change behavior. More specifically, when the context in which a behavior is habitually performed is subject to change, the environmental cues that automate behavior are no longer present and there is an increased likelihood that individuals will re-consider their behavior and adopt alternatives (e.g., walking to school). Indeed, interventions delivered shortly before, during or after a context change have been shown to be effective at changing habitual behavior [54]. In the present context, interventions promoting walking to school might usefully be delivered during the transition phase from elementary to high school, given that this context change broadly corresponds with the development phase in which physical activity notably decreases (i.e., the transition from childhood to early adolescence). Also, on the basis that many parents do not permit their children to walk to school, these interventions might also need to be targeted at parents/guardians. More generally, it is likely that interventions designed to support active school travel habits will need to be sustained over long periods of time (i.e., until the required behavior becomes automated).

### Potential limitations

Although this study provides valuable insights into children’s active school travel and has important implications for the development and delivery of interventions, the findings need to be interpreted in light of the potential limitations of the study. First, all participants were sampled from schools in one urban region of Scotland. Future research is needed to test the extent to which the findings generalize to children living in other (e.g., rural) areas. Second, the data were collected during the fall school term and there is seasonal variation in children’s school travel behavior, with walking to school being less prevalent during fall and winter compared with spring or summer [48]. However, replicating this study in the spring or summer would only be expected to provide stronger evidence for the predictive validity of the TPB and habit on the basis that there is greater variation in active travel behavior during this time of year. Third, the length of the follow up period used in the present study was just one week, meaning that only short-term effects were investigated. That said, previous research has shown considerable stability in children’s school travel behavior over longer periods of time [5]. Even so, it would be worth testing the effects of TPB constructs and habit using a longer follow-up period than used in the present study.

## Conclusion

Overall, the present study provides support for the application of the TPB to children’s active school travel behavior (walking to school). Additionally, in line with expectations, habit strength augmented the predictive validity of the model. The findings imply that children’s active school travel is underpinned by both reasoned and habitual processes. Further research is needed to identify effective techniques for changing the predictors of children’s active school travel identified in the current study.

## Endnotes

^1^ Habit was operationalized with respect to both walking and car/bus use on the basis that approximately half of children in the present age range walk to school and approximately half travel by car or bus (National Travel Survey, 2009). Thus, given that school travel is almost equally distributed between these two modes, habituation with respect to both modes has the potential to impact on active school travel.

## Competing interests

The authors declare that they have no competing interests.

## Authors' contributions

DAR, NMN, DM and SM designed the study and wrote the protocol. SM, DM and DAR managed the data collection phase. SM and MAE conceptualised the research question and carried out analyses and interpreted the data. The writing of the manuscript was led by SM with the assistance of MAE. All authors provided additional comments on the final draft and have read and approved the final version of the manuscript. The study was conducted as part of SM’s PhD thesis, under the supervision of DAR, NMN and MAE.

## References

[B1] Andersen LB Harro M Sardinha LB Froberg K Ekelund U Brage S Anderssen SA Physical activity and clustered cardiovascular risk in children: A cross-sectional study (The European Youth Heart Study) Lancet 2006 368 299 304 10.1016/S0140-6736(06)69075-2 16860699

[B2] Riddoch CJ Leary SD Ness AR Blair SN Deere K Mattocks C Griffiths A Smith GD Tilling K Prospective associations between objective measures of physical activity and fat mass in 12-14 year old children: The Avon Longitudinal Study of Parents and Children (ALSPAC) BMJ 2006 339 b4544 19942665 10.1136/bmj.b4544 PMC2784494

[B3] Sibley BA Etnier JL The relationship between physical activity and cognition in children: A meta-analysis Pediatr Exerc Sci 2003 15 243 256

[B4] Currie C Levin K Kirby J Currie D van der Sluijs W Inchley J Health Behaviour in School-aged Children (HBSC): World Health Organization Collaborative Cross-National Study: Findings from the 2010 HBSC survey in Scotland 2011 The University of Edinburgh, Child and Adolescent Health Research Unit

[B5] Inchley J Kirby J Currie C Physical Activity in Scottish Schoolchildren (PASS) Project: Physical activity among adolescents in Scotland: final report of the PASS study 2008 The University of Edinburgh, Child and Adolescent Health Research Unit

[B6] Telama R Yang X Viikari J Vãlimãki I Wanne O Raitakari O Physical activity from childhood to adulthood. A 21-year tracking study Am J Prev Med 2005 28 267 273 10.1016/j.amepre.2004.12.003 15766614

[B7] Chillón P Evenson KR Vaughn A Ward DS A systematic review of interventions for promoting active transportation to school Int J Behav Nutr Phys Act 2011 8 10 10.1186/1479-5868-8-10 21320322 PMC3050785

[B8] Verplanken B Agnew CR, Calston DE, Graziano WG, Kelly JR Habit: from overt action to mental events Then a miracle occurs: Focusing on behaviour in social psychology theory and research 2010 Oxford University Press, Oxford 68 88

[B9] Ajzen I The theory of planned behavior Organ Behav Hum 1991 50 179 211 10.1016/0749-5978(91)90020-T

[B10] Sutton S Rutter D, Quine L Using social cognition models to develop health behaviour interventions: problems and assumptions Changing Health Behaviour 2002 Open University Press, Buckingham 108 193

[B11] Elliott MA Thomson JA Social cognitive determinants of offending drivers’ speeding behaviour Accid Anal Prev 2010 42 1595 1605 10.1016/j.aap.2010.03.018 20728608

[B12] Cialdini RB Reno RR Kallgren CA A focus theory of normative conduct: Recycling the concept of norms to reduce littering in public places J Perso Soc Psychol 1990 58 6 1015 1026

[B13] Sheeran P Trafimow D Armitage CJ Predicting behaviour from perceived behavioural control: A test of the accuracy assumption of the theory of planned behaviour Brit J Soc Psych 2003 42 393 410 10.1348/014466603322438224 14567844

[B14] Faulkner GE Buliung RN Flora PK Fusco C Active school transport, physical activity levels and body weight of children and youth: A systematic review Prev Med 2009 48 1 3 8 10.1016/j.ypmed.2008.10.017 19014963

[B15] Gardner B Abraham C Psychological correlates of car use: A meta-analysis Transport Res F: Traffic Psychol Behav 2008 11 300 311 10.1016/j.trf.2008.01.004

[B16] Hagger MS Chatzisarantis NLD Biddle SJH A meta-analytic review of the theories of reasoned action and planned behavior in physical activity: Predictive validity and the contribution of additional variables J Sport Exerc Psychol 2002 24 3 32

[B17] Armitage C Conner M Efficacy of the theory of planned behaviour: A meta-analytic review Brit J Soc Psychol 2001 40 471 499 10.1348/014466601164939 11795063

[B18] McEachan RRC Conner M Taylor NJ Lawton RJ Prospective prediction of health-related behaviours with the theory of planned behaviour: a meta-analysis Health Psychol Rev 2011 in press manuscript

[B19] Cohen JL A power primer Psychol Bull 1992 112 155 159 19565683 10.1037//0033-2909.112.1.155

[B20] Downs DS Hausenblas HA The theories of reasoned action and planned behavior applied to exercise: A meta-analytic update J Phys Act Heal 2005 2 76 97

[B21] Rhodes RE Plotnikoff RC Can current physical activity act as a reasonable proxy measure of future physical activity? Evaluating cross-sectional and passive prospective designs with the use of social cognition models Prev Med 2005 40 547 555 10.1016/j.ypmed.2004.07.016 15749137

[B22] Armitage CJ Reid JC Spencer CP Changes in cognition and behaviour: A causal analysis of single-occupancy car use in a rural community Transportmetrica 2011 In press 10.1080/18128602.2010.509706

[B23] Luchins AS Primacy-recency in impression formation 1957 Yale University Press, In The order of presentation in persuasion Edited by Hovland C, New Haven, CN 33 61

[B24] Bower GH How emotions might affect learning? In The handbook of emotion and memory: Research and theory Edited by Christianson SA 1992 Lawrence Erlbaum Associates, Hillsdale, NJ 3 31

[B25] Paulhus DL Socially desirable responding: The evolution of a construct 2002 Lawrence Erlbaum Associates, In The role of constructs in psychological and educational measurement Edited by Braun H, Jackson DN, Wiley DE, Hillsdale, NJ 67 88

[B26] Rowe DA Mahar MT Raedeke TD Lore J Measuring physical activity in children with pedometers: reliability, reactivity, and replacement of missing data Pediatr Exerc Sci 2004 16 343 354

[B27] Adamo K Prince SA Tricco A Connor-Gorber S Tremblay M A comparison of indirect versus direct measures for assessing physical activity in the pediatric population: a systematic review Int J Pediatr Obes 2009 4 2 27 10.1080/17477160802315010 18720173

[B28] Elliott MA Armitage CJ Baughan CJ Using the theory of planned behaviour to predict observed driver behaviour Brit J Soc Psych 2007 46 69 90 10.1348/014466605X90801 17355719

[B29] Rhodes RE MacDonald HM McKay HE Predicting physical activity intention and behaviour among children in a longitudinal sample Soc Sci Med 2006 62 3146 3156 10.1016/j.socscimed.2005.11.051 16406632

[B30] Martin JJ Oliver K McCaughtry N The theory of planned behavior: Predicting physical activity in Mexican American children J Sport Exerc Psychol 2007 29 225 238 17568068 10.1123/jsep.29.2.225

[B31] Ouellette JA Wood W Habit and intention in everyday life: the multiple processes by which past behavior predicts future behavior Psychol Bull 1998 124 54 74

[B32] De Bruijn GJ Understanding college students’ fruit consumption. Integrating habit strength in the theory of planned behaviour Appetite 2010 54 1 16 22 10.1016/j.appet.2009.08.007 19712718

[B33] De Bruijn GJ Kroeze W Oenema A Brug J Saturated fat consumption and the Theory of Planned Behaviour: Additive and interaction effects of habit strength Appetite 2008 51 318 323 10.1016/j.appet.2008.03.012 18471932

[B34] Kremers SPJ Dijkman MAM de Meij JSB Jurg ME Brug J Awareness and habit: Important factors in physical activity in children Health Education 2008 108 6 475 488 10.1108/09654280810910881

[B35] Norman P Cooper Y The theory of planned behaviour and breast self-examination: Assessing the impact of past behaviour, context stability and habit strength Psychol Health 2011 26 1156 1172 10.1080/08870446.2010.481718 21391130

[B36] Murtagh S Rowe DA McMinn D Nelson NM Reliability and validity of a measure of active and inactive travel habit in primary school aged children. Presented at the British Association of Sport and Exercise Sciences Annual Conference 2010 Glasgow, Scotland

[B37] Kremers SPJ Dijkman MAM de Meij JSB Jurg ME Brug J Awareness and habit: Important factors in physical activity in children Heal Educ 2008 108 6 475 488 10.1108/09654280810910881

[B38] Wind M Kremers S Thijs C Brug J Toothbrushing at school: Effects on toothbrushing behaviour, cognitions and habit strength Health Education 2005 105 1 53 61 10.1108/09654280510572303

[B39] Norman P The theory of planned behavior and binge drinking among undergraduate students: Assessing the impact of habit strength Addict Behav 2011 36 502 507 10.1016/j.addbeh.2011.01.025 21310540

[B40] Ajzen I Residual effects of past on later behavior: Habituation and reasoned action perspectives Perso Soc Psychol Rev 2002 6 107 122 10.1207/S15327957PSPR0602_02

[B41] Aarts H Dijksterhuis A Habits as knowledge structures: Automaticity in goal-directed behavior J Pers Soc Psychol 2000 78 53 63 10653505 10.1037//0022-3514.78.1.53

[B42] Evenson KR Catellier DJ Gill K Ondrak KS McMurray RG Calibration of two objective measures of physical activity for children J Sports Sci 2008 26 14 1557 1565 18949660 10.1080/02640410802334196

[B43] Mattocks C Leary S Ness A Deere K Saunders J Tilling K Kirkby J Blair SN Riddoch C Calibration of an accelerometer during free-living activities in children Int J Pediatr Obes 2007 2 4 218 226 10.1080/17477160701408809 17852552

[B44] Ozdoba R Corbin C Le Masurier G Does reactivity exist in children when measuring activity levels with unsealed pedometers? Pediatr Exerc Sci 2004 16 158 166

[B45] Fishbein M Ajzen I Predicting and changing behavior: The reasoned action approach 2010 Psychology Press, New York, NY

[B46] Verplanken B Orbell S Reflections on past behavior: A self-report index of habit strength J App Soc Psychol 2003 33 1313 1330 10.1111/j.1559-1816.2003.tb01951.x

[B47] Godin G Kok G The theory of planned behavior: A review of its applications in health-related behaviors Am J Promo 1996 11 87 98 10.4278/0890-1171-11.2.87 10163601

[B48] Granville S Laird A Barber M Rait F Why do Parents Drive their Children to School? 2006 Transport Research Series, Edinburgh, Scottish Executive Central Research Unit

[B49] Sallis JF Prochaska JJ Taylor WC A review of correlates of physical activity of children and adolescents Med Sci Sports Exerc 2000 32 963 975 10795788 10.1097/00005768-200005000-00014

[B50] Bandura A Self-efficacy: The exercise of control 1997 New York, Freeman

[B51] Fencl HS Scheel KR Engaging students: an examination of the effects of teaching strategies on self-efficacy and course climate in a nonmajors physics course J Coll Sci Teach 2005 35 20 25

[B52] Margolis H McCabe P Motivating struggling readers in an era of mandated instructional practices Read Psychol 2006 27 435 455 10.1080/02702710600848023

[B53] Verplanken B Walker I Davis A Jurasek M Combining the habit discontinuity and self-activation hypotheses in explaining travel mode choices J Environ Psychol 2008 28 2 121 127 10.1016/j.jenvp.2007.10.005

[B54] Bamberg S Is a Residential Relocation a Good Opportunity to Change People's Travel Behavior? Results From a Theory-Driven Intervention Study Environ Behav 2006 38 820 840 10.1177/0013916505285091

